# Lanolin and Its Imitations

**Published:** 1888-09

**Authors:** 


### LANOLIN AND ITS IMITATIONS.

German medical literature contains frequent testimonials from clinical observers of the extended uses of lanolin as an unguent basis in the treatment of skin diseases, the therapeutic application of inunction to the treatment of constitutional diseases, as an obstetric application in the treatment of wounds and cuts, and cosmetically in the prevention of sunburn and freckling, and even the "prevention of wrinkles." Professor Oscar Liebriech's most valuable and original addition to our storehouse of therapeutic resources is, however, subject to the usual drawback which attends the introduction of any novel and useful substance, by the occasional substitution of imperfect imitations and inferior products simulating lanolin, at least in title, which produce deleterious effects. Dr. G. Meyer, of Berlin, reports to the following effect in the *Deutsche Med. Wochenschr.*, No. 19, 1888: A man, aged 38, had a hard chancre in 1875, and was treated in the Charité Hospital, Berlin, and elsewhere for relapses, during the next five years. In June of last year he suffered from hoarseness and swelling of the testicles. Amongst other treatment, the following ointment was applied to the testicles: pot. iodid. 1 gramme; lanolin, 10 grammes. The word "lanolin" was written without any affix. Severe acute dermatitis of the scrotum followed, with great swelling, redness, and burning heat of the skin. The penis participated. The man could not walk about, and, therefore, left off work. The inflammation, he said, set in after the fourth infriction. The general health was not affected; the penis, as above said, shared in the inflammation, which affected both sides of the scrotum. Epididymitis was excluded accordingly, and suspicion rested on the ointment. More of this was ordered as before, for examination only, and it smelt "acid and rancid." The skin remained like thick leather for some time. An ointment of one part of potassium iodide in ten parts of lanolin puriss. (Liebreich) was then rubbed in by Dr. Meyer himself. The skin was distinctly thinner two days afterwards, without any trace of irritation by the ointment. The previous inflammation was due, there is no doubt, to the rancid lanolin used, for all sorts of lanolin, except Liebreich's, contain several fatty acids. The percentages of these in various samples of lanolin are given in the *Deutsche Med. Wochenschr.*, No. 28, 1886, as 33, 26, 17, 16, 16; in Jaffé and Darmstaedter's lanolin, 0.5 to 1.—*The British Medical Journal.*

